# Editorial: Child sexual exploitation: a cross-cultural analysis of risk factors and protective strategies

**DOI:** 10.3389/fpubh.2026.1861478

**Published:** 2026-06-16

**Authors:** Jane Reeves, Aravinda Kosaraju, Stephanie Jones

**Affiliations:** 1 Centre for Child Protection, School of Social Sciences, University of Kent, Canterbury, United Kingdom; 2 Independent Researcher, Brisbane, QLD, Australia

**Keywords:** child sexual exploitation and abuse, protective strategies, risk and harm prevention, safeguarding children, systemic approaches

We live in unprecedented times of child sexual exploitation and abuse (CSEA), and the interconnected systems, stakeholders, and environments of the digital ecology have rendered its ubiquity complex. Globally, one in five girls and one in 13 boys have been sexually abused or exploited before they are 18 years old (UNICEF 2021). Effective protection of children from CSEA, both online and offline, requires collaborative governance at all levels of our social ecology and ought to be underpinned by child-centered approaches. This special edition on “*Child sexual exploitation: a cross-cultural analysis of risk factors and protective strategies*” reflects the complexity of CSEA, brings together a variety of pioneering solutions and preventative methods, and underscores the importance of a multifaceted approach to child safety that combines parental involvement, technological innovation, education, and policy reform.

Addressing the macro-, meso-, and micro-systemic issues of this omnipresent problem, the articles in this journal, taken together, indicate the emergence of potential solutions. At the macro level, the article by Young et al. uses a case study methodology to examine how cultural beliefs are integrated into CSEA risk assessments and policy responses in three different cultural contexts: Malaysia, Indonesia, and Brunei Darussalam. It also offers reflection points for policymakers, when conceptualizing legislative and policy responses to CSEA, to consider how cultural beliefs may impact the development or implementation of responses. Moreover, the article by Nwuzoh et al. examines the age assurance framework developed within the UK's regulatory architecture and its transferability as a mechanism to safeguard children in Sub-Saharan Africa. Guided by the Health Policy Triangle, they note five policy design elements that are relevant whilst taking into consideration equity and capacity constraints that are context-specific.

At the meso level of the ecosystem, three articles examine the interplay between technology and preventative strategies. Rivera-Rivera et al. present a protocol for a direct intervention with school children using immersive reality to prevent CSEA in Mexico, with the potential to enhance elementary school children's knowledge of self-esteem, body safety, and their rights, helping them prevent child violence in Mexico. Although the article by Green et al. focuses on child-centered policing that is trauma-informed when working with girls with lived experience of child sexual exploitation, it reports the results of a pilot training programme using a digitally simulated training tool called “Robyn and Molly,” with demonstrable improvements in officers' self-reported confidence, knowledge, and practice skills in identifying child sexual exploitation, supporting victims, and investigating crimes in a trauma-informed manner. It also indicates reductions in victim-blaming language and greater use of trauma-informed framing in cases involving the sexual exploitation of children. The final article exploring another meso-level intervention, by Davy et al., adopts a user-centered design approach to develop an “on-device” technology to prevent the viewing of child sexual abuse material, focusing on potential perpetrators of online CSEA. Based on the findings, the authors propose seven evidence-based design principles for user-centered, on-device harm-reduction technology. Additionally, in their systematic review, Punjani et al. highlight the complex intersection of micro and macro level barriers that hinder effective parental engagement in preventing CSEA online and call for more holistic approaches that support parental efforts to curb sexual abuse and exploitation of children online.

Finally, at the micro level, the article by Godoy et al. focuses on family dynamics and examines the complexities of young people engaging in high-risk sexual behaviors. Their research debunks the commonly held assumption that family relationships are protective against child sexual exploitation and highlights the heterogeneity of family circumstances and adverse childhood experiences among sexually active adolescents who have experienced child sexual exploitation or other sexual risk factors. This article reiterates the need to understand the considerable complexity of how family functioning relates to child sexual exploitation. This has implications for practice, suggesting that interventions should take into account peer microsystems and be tailored to the contexts and times that shape young people's experiences.

This special edition captures a wide scope of interventions and focuses on innovative solutions that target differing and overlapping societal systems. In doing so, the included studies also draw on a range of different methodologies, including secondary data analysis, case studies, participatory action research, and randomized controlled trials. This is significant because the breadth and diversity of research represented in this special edition reflect the layered and complex nature of CSEA and underscore the need to address the issue using multiple approaches.

In this ever-changing world, crimes involving children continue to take new forms, many of which now involve digital technologies. Evolving technology will continue to challenge the types of solutions needed to help children and professionals tackle this. This is why it is more important than ever to continue developing preventative education content and equipping all levels of society surrounding the child, as well as the child themselves, with the skills to identify risks, safeguard against unsafe situations, and respond appropriately. Although there is no single answer to the question of how to safeguard children from sexual abuse and exploitation, it is clear that there is consensus on the need to uphold the right of the child to be protected from sexual exploitation (Article 34 of the UNCRC) and to recover from trauma (Article 39 of the UNCRC), across every continent and within the digital ecosystem. Whether at home, in school, within health and political systems, across policing and policy systems, or on online platforms and in the technology space, safe spaces must exist wherever a child is present. Far more than anything else, there is significance in the inclusion of the child's voice in these matters, a fundamental child right in its own right (Article 12 of the UNCRC). Indeed, when we include children in the conversation, we create space for stronger, more holistic solutions.

